# Artificial intelligence prediction model for overall survival of clear cell renal cell carcinoma based on a 21-gene molecular prognostic score system

**DOI:** 10.18632/aging.202594

**Published:** 2021-03-03

**Authors:** Qiliang Peng, Yi Shen, Kai Fu, Zheng Dai, Lu Jin, Dongrong Yang, Jin Zhu

**Affiliations:** 1 Department of Radiotherapy and Oncology, The Second Affiliated Hospital of Soochow University, Suzhou, China; 2 Institute of Radiotherapy and Oncology, Soochow University, Suzhou, China; 3 Department of Radiation Oncology, The Affiliated Suzhou Science and Technology Town Hospital of Nanjing Medical University, Suzhou, China; 4 Department of Urology, The Second Affiliated Hospital of Soochow University, Suzhou, China

**Keywords:** clear cell renal cell carcinoma, prognosis, artificial intelligence, scoring system

## Abstract

We developed and validated a new prognostic model for predicting the overall survival in clear cell renal cell carcinoma (ccRCC) patients. In this study, artificial intelligence (AI) algorithms including random forest and neural network were trained to build a molecular prognostic score (mPS) system. Afterwards, we investigated the potential mechanisms underlying mPS by assessing gene set enrichment analysis, mutations, copy number variations (CNVs) and immune cell infiltration. A total of 275 prognosis-related genes were identified, which were also differentially expressed between ccRCC patients and healthy controls. We then constructed a universal mPS system that depends on the expression status of only 21 of these genes by applying AI-based algorithms. Then, the mPS were validated by another independent cohort and demonstrated to be applicable to ccRCC subsets. Furthermore, a nomogram comprising the mPS score and several independent variables was established and proved to effectively predict ccRCC patient prognosis. Finally, significant differences were identified regarding the pathways, mutated genes, CNVs and tumor-infiltrating immune cells among the subgroups of ccRCC stratified by the mPS system. The AI-based mPS system can provide critical prognostic prediction for ccRCC patients and may be useful to inform treatment and surveillance decisions before initial intervention.

## INTRODUCTION

Renal cell carcinoma (RCC) is one of the most lethal cancer types in the urinary system, which accounts for 85%-95% of renal malignancies and 2%-3% of all human tumors [1]. Clear cell renal cell carcinoma (ccRCC) is the most common subtype of RCC, accounting for approximately 70%-85% of the pathological types of cases [2]. In the past three decades, the incidence of ccRCC has increased gradually and approximately 30% patients already have metastasis when they were first diagnosed with ccRCC. Currently, treatments for localized ccRCC mainly depend on radio-frequency ablation and partial or radical nephrectomy; nevertheless, when ccRCC progresses to distant metastasis, the curative effect of present targeted drug therapies is unsatisfactory [3]. To this end, it is necessary to identify accurate predictors in ccRCC that are helpful for detecting it early, monitoring tumor progression, revealing survival outcome, promoting personalized therapy and for the purpose of individualized follow-up strategies.

Currently, the American Joint Committee on Cancer (AJCC) tumor node metastasis (TNM) staging system and Fuhrman grade have been the most widely accepted clinical classification systems for prognostic prediction of ccRCC. In addition, the University of California Integrated Staging System (UCISS) and stage, size, grade, and necrosis (SSIGN) score are also applied [4]. However, ccRCC patients with the same clinical stage can still have different survival outcomes, suggesting that these methods cannot accurately evaluate and manage patients with ccRCC [5]. Recent rapid advances in research techniques have promoted the development of various molecular prognostic indicators [6]. A series of genetic or epigenetic alterations have been demonstrated to play a vital role in the initiation and progression of ccRCC and may serve as potential biomarkers for clinical application [7]. Nevertheless, none of the tests developed to date are sufficiently qualified to predict overall survival. For this reason, a more effective and comprehensive tool for understanding of the molecular classification characteristics of ccRCC is urgently required.

Artificial intelligence (AI) has been known as a field of science and engineering involved in the computational understanding of intelligent behavior such as perceiving one’s environment, acting in complex environments, learning and understanding from experience, using reasoning to solve problems and to discover hidden knowledge, and applying knowledge successfully in new situations [8]. Machine learning, a branch of AI technology, has been recognized as a collection of data-analytical techniques aimed at building predictive models from multi-dimensional datasets [9]. With the utilization of AI-based machine learning method, new data could be predicted based on the identified patterns through detecting difficult-to-recognize patterns from complex combinations of multiple biomarkers [10]. Based on these advantages, AI-based machine learning has gained great attention and has been widely applied in medical fields for image detection, diagnosis, and outcome prediction [11].

In the present study, we created a system applying AI-based machine learning approaches and applied it to establish a universal molecular prognostic score (mPS) for overall survival prediction of ccRCC that relies on the expression status of 21 genes. During the construction of mPS, we applied two prediction models, a random forest prediction model and an artificial neural network model, for predicting the survival outcome of ccRCC. We then validated the mPS system by using different patient cohorts. Afterwards, a comprehensive nomogram comprising the mPS system and several independent variables were set up to predict ccRCC patient survival. In addition, we compared the differences among three subgroups of ccRCC samples, which were divided according to mPS scores, regarding mutations and copy number variations (CNVs), and immune cell infiltrations.

## RESULTS

### Differentially expressed genes related to the prognosis of ccRCC

**Figure 1:**
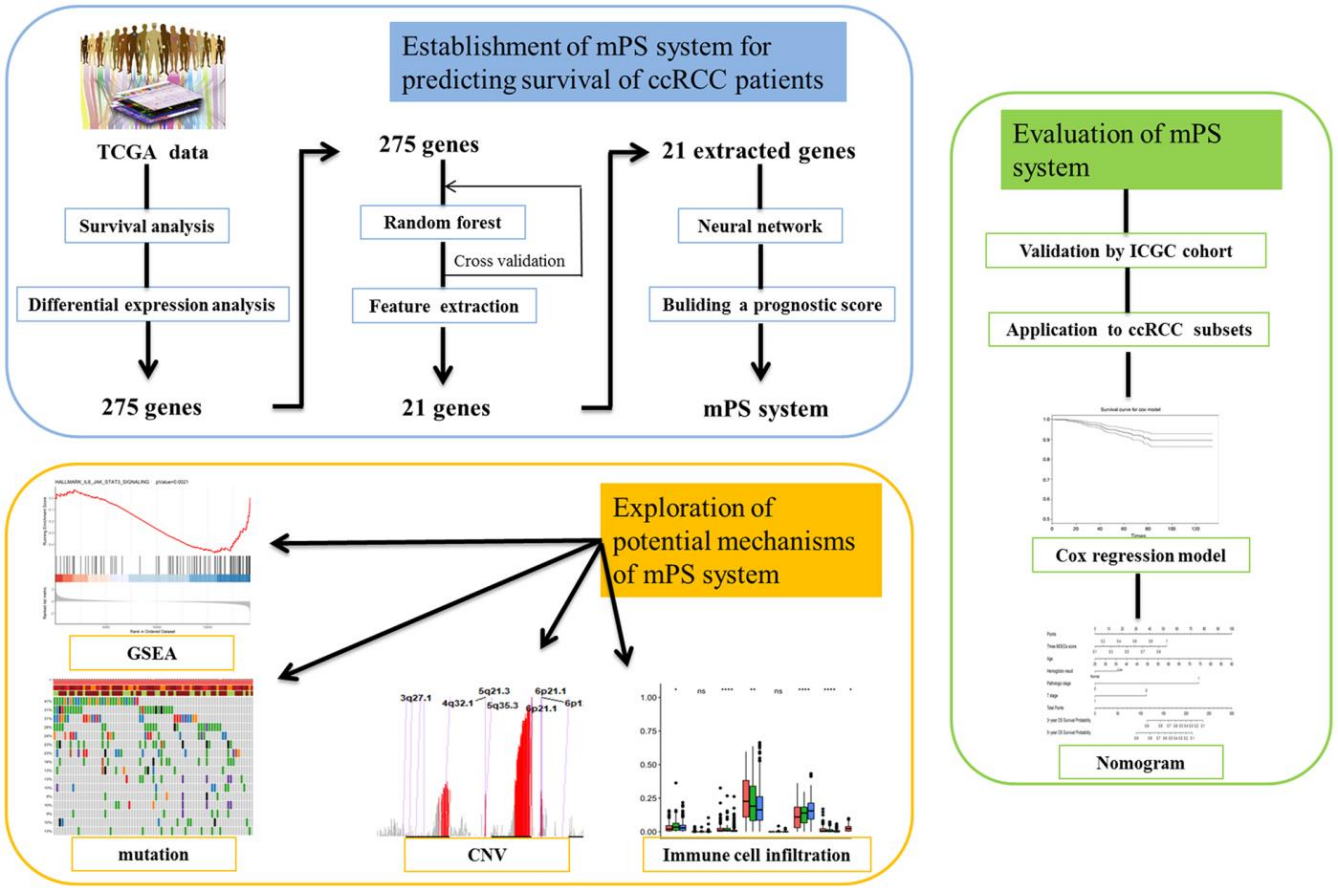
**Study pipeline.** In this study we first used the TCGA clear cell renal cell carcinoma (ccRCC) cohort to identify a list of 275 genes, which were associated with the prognosis of ccRCC patients according to survival analysis and were also differentially expressed between ccRCC patients and healthy controls. Then, artificial intelligence (AI) methods including random forest and neural network were applied to establish the mPS system based on 21 prognosis-related genes. Then, we validated the mPS system by using the ICGC cohort. Next, we found that the mPS system could be applied to ccRCC subsets. Moreover, we evaluated the mPS system by conducting univariate and multivariate Cox regression analysis of the TCGA dataset and built a nomogram comprising the mPS score and several independent variables to predict ccRCC patient prognosis. Finally, we explored the potential mechanisms underlying the mPS system by performing gene set enrichment analysis (GSEA), mutations, copy number variations (CNVs) and immune cell infiltration analysis.

**Figure 2:**
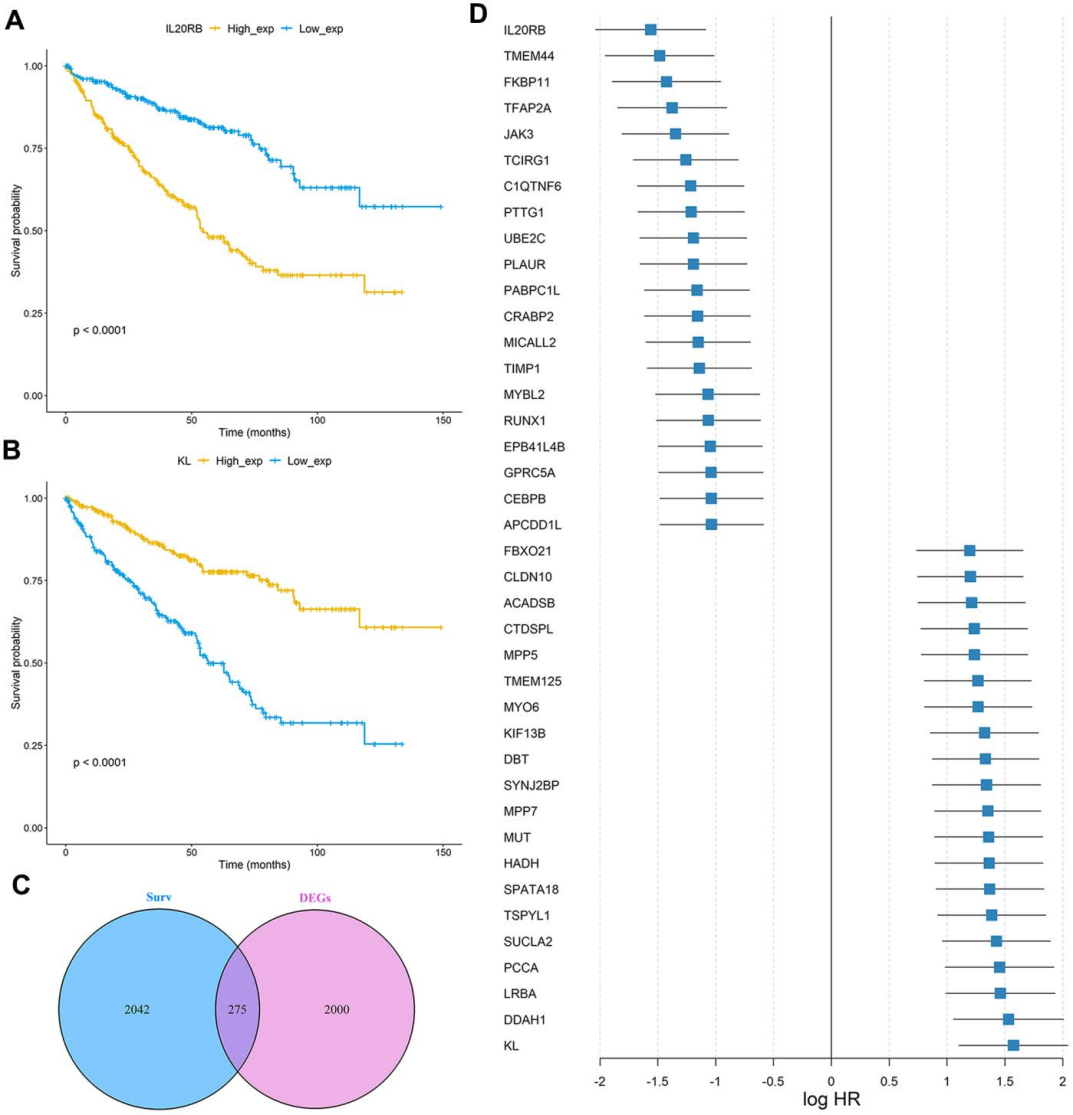
**Identification of prognosis-related genes in the TCGA ccRCC cohort.** (**A**, **B**) Kaplan-Meier curves of OS for the TCGA cohort based on IL20RB and KL expression levels, respectively; (**C**) Venn diagram of prognostic-related genes and differentially expressed genes in TCGA cohort; (**D**) The integrated HR for Top 40 prognosis-related genes in the TCGA data sets.

The study design was plotted in Figure 1. To study the specific genes associated with the prognosis in patients with ccRCC, we downloaded gene expression data from 531 ccRCC samples with clinical survival information from The Cancer Genome Atlas (TCGA) database. The detailed characteristics are presented in Table 1. We divided the 531 ccRCC samples into a high expression group and a low expression group according to the median expression of genes, and then observed the relationships between gene expression level and overall survival (OS) in these two groups. A total of 2317 OS-related genes in the TCGA discovery cohort were identified including Interleukin 20 Receptor Subunit Beta (IL20RB) and KL (Klotho) (Figure 2A, 2B). Then the gene differential expression analysis was conducted on 531 ccRCC samples and 72 healthy control samples in TCGA. Based on the Limma R package, a total of 2275 genes were identified to differentially express between ccRCC patients and healthy controls. After intersection with the prognosis-related genes and differential expressed genes, a total of 275 differentially expressed genes which were associated with the prognosis of ccRCC were retained (Figure 2C). In particular, IL20RB and KL were the most promising prognosis-related genes, with the lowest and highest hazard ratios (HRs), respectively, in the TCGA discovery cohort. The integrated HR for top 40 prognosis related genes in the TCGA data sets were plotted at Figure 2D.

**Table 1:** TCGA cohort ccRCC patient characteristics.

**Clinical characteristic**	**Variable**	**Total**	**Percentages (%)**
Age (years)	>60	266	50.09
	≤60	265	49.91
Gender	Female	186	35.03
	Male	345	64.97
Laterality	Left	250	47.08
	Right	280	52.73
	Others	1	0.19
Histological grade	G1	14	2.64
	G2	227	42.75
	G3	207	38.98
	G4	75	14.12
	GX	5	0.94
	Unknown	3	0.56
T	T1	272	51.22
	T2	69	12.99
	T3	179	33.71
	T4	11	2.07
N	N0	239	45.01
	N1	16	3.01
	NX	276	51.98
M	M0	420	79.10
	M1	78	14.69
	MX	31	5.84
	Unknown	2	0.38
Pathological stage	Stage I	266	50.09
	Stage II	57	10.73
	Stage III	123	23.16
	Stage IV	82	15.44
	Unknown	3	0.56
Survival status	Alive	359	67.61
	Dead	172	32.39

### AI-based development of the mPS system

**Figure 3:**
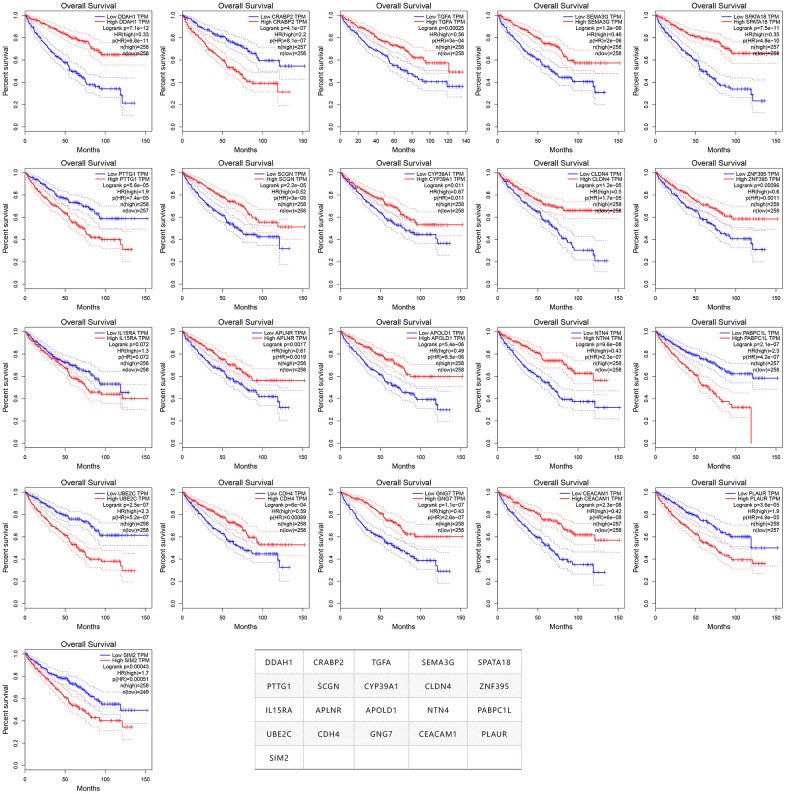
**The survival curves of the 21 prognosis-related genes used for constructing the mPS system.**

In order to verify whether these 275 genes are enough to predict the survival rate of ccRCC patients at 3 years, we used the TCGA ccRCC cohort to construct a molecular prognosis score system as illustrated in detail in the Materials and methods. We first used the random forest algorithm for feature gene screening. When the cutoff of feature importance was set to 0.5, a total of 21 genes were identified and then used for the following artificial neural network construction. The survival curves of the 21 genes were illustrated at Figure 3.

**Figure 4:**
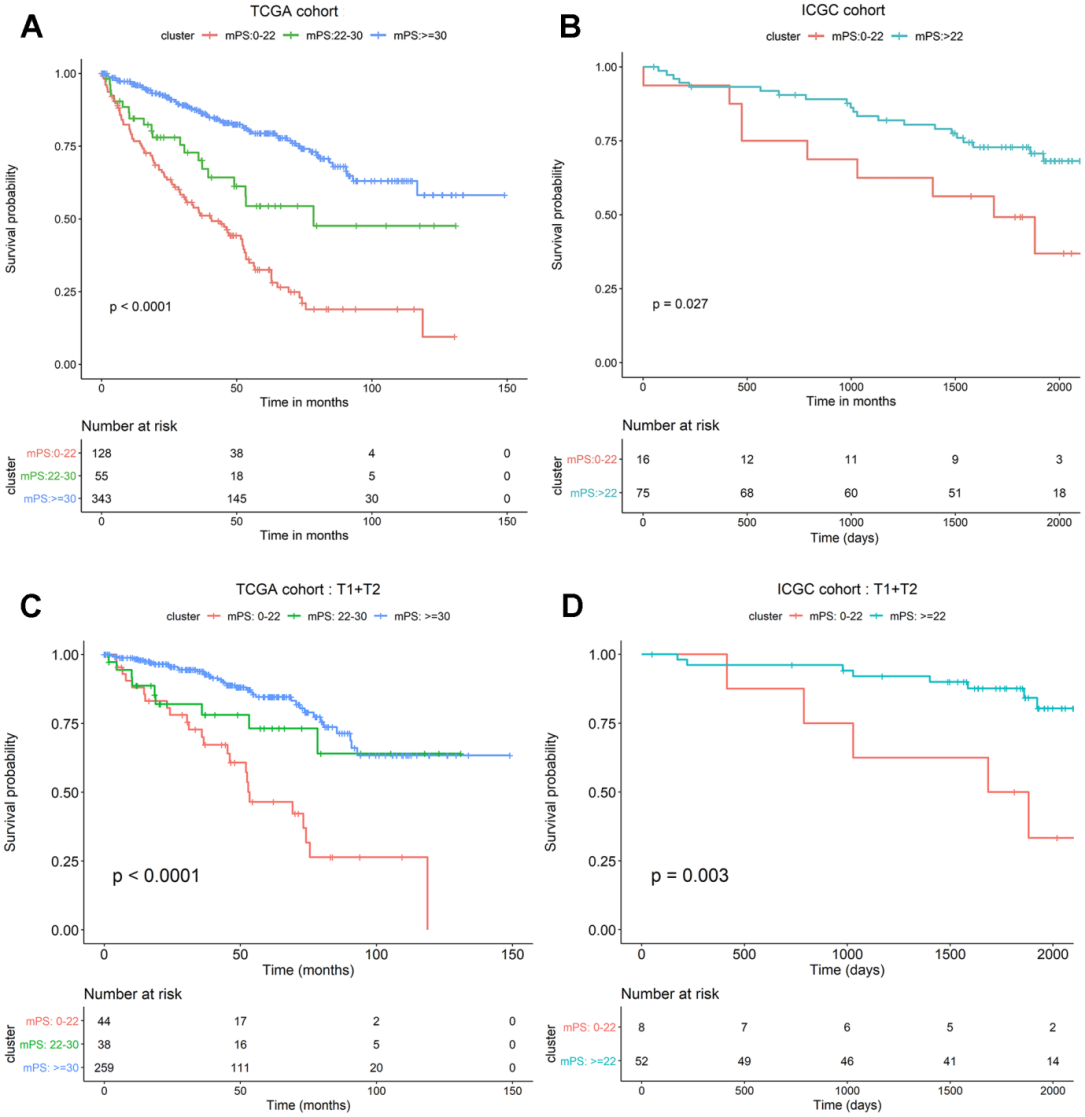
**mPS system can precisely stratify prognosis of ccRCC patients and is applicable to T1 and T2 ccRCC subsets.** (**A**) Kaplan-Meier curves of OS according to mPS for the TCGA cohort; (**B**) Kaplan-Meier curves of OS according to mPS for the ICGC cohort; (**C**) Kaplan-Meier curves according to mPS for OS of patients at clinical T1 and T2 stage in the TCGA cohort; (**D**) Kaplan-Meier curves according to mPS for OS of patients at clinical T1 and T2 stage in the ICGC cohort.

The neural network model consisted of an input layer, two hidden layers and an output layer. The 21 genes were used as the input layer, and the middle 2 hidden layers included 4 neurons and 2 neurons, respectively. The output layer used the softmax activation function and consisted of two dimensions, each of which represented an outcome: alive or deceased. Based on the constructed neural network model, we obtained the weight of each gene, and then built a mPS system, which was measured by summation of “Gene-Score” × “Gene-Weight” for all 21 genes. For the TCGA cohort, the mPS value ranges from 0 to 71.57 (Table 2). Under each mPS value, the samples were stratified into three groups: low-mPS (mPS < 22), median-mPS (22 ≤ mPS < 30), and high-mPS group (mPS ≥ 30). Then, survival analysis was conducted with the Kaplan-Meier (K-M) method and log-rank statistical test. The results indicated that there was a significant difference among the three groups (Figure 4A).

**Table 2:** The 21 genes necessary and sufficient for calculation of mPS.

**Symbol**	**Gene ID**	**Full name**	**Score** **(high)**	**Score** **(low)**	**Weight**
DDAH1	23576	Dimethylarginine dimethylaminohydrolase 1	1	0	3.580
CRABP2	1382	Cellular retinoic acid binding protein 2	0	1	3.009
TGFA	7039	Transforming growth factor alpha	1	0	3.425
SEMA3G	56920	Semaphorin 3G	1	0	3.042
SPATA18	132671	Spermatogenesis associated 18	1	0	4.169
PTTG1	9232	Pituitary tumor-transforming gene 1	0	1	3.344
SCGN	10590	Secretagogin	1	0	4.200
CYP39A1	51302	Cytochrome P450 family 39 subfamily A member 1	1	0	3.595
CLDN4	1364	Claudin 4	1	0	3.541
ZNF395	55893	Zinc finger protein 395	1	0	3.977
IL15RA	3601	Interleukin 15 receptor subunit alpha	0	1	3.859
APLNR	187	Apelin receptor	1	0	3.072
APOLD1	81575	Apolipoprotein L domain containing 1	1	0	3.207
NTN4	59277	Netrin 4	1	0	3.723
PABPC1L	80336	Poly(A) binding protein cytoplasmic 1 like	0	1	3.670
UBE2C	11065	Ubiquitin conjugating enzyme E2 C	0	1	4.217
CDH4	1002	Cadherin 4	1	0	3.183
GNG7	2788	G protein subunit gamma 7	1	0	3.515
CEACAM1	634	CEA cell adhesion molecule 1	1	0	3.506
PLAUR	5329	Plasminogen activator, urokinase receptor	0	1	3.418
SIM2	6493	SIM bHLH transcription factor 2	0	1	3.364

### Validation of the mPS system in independent cohorts

To assess whether mPS is feasible for the prediction of prognosis in other independent ccRCC cohorts, we investigated another ccRCC data set from International Cancer Genome Consortium (ICGC, https://icgc.org/). The detailed characteristics of ICGC cohort are presented in Table 3. We first calculated the mPS score of each sample in the ICGC data, and then divided the samples into different groups according to the above mentioned methods. Since the sample size of ICGC database is too small, we only divided the ICGC dataset into two groups: low-mPS (mPS < 22) and high-mPS (mPS ≥ 22) groups. The results revealed that there was a significant difference about the prognosis among different mPS groups. These findings demonstrated that mPS can stratify prognosis in different ccRCC cohorts (Figure 4B).

**Table 3:** ICGC cohort ccRCC patient characteristics.

**Clinical characteristic**	**Variable**	**Total**	**Percentages (%)**
Age (years)	>60	45	49.45
	≤60	46	50.55
Gender	Female	39	42.86
	Male	52	57.14
Status	Alive	61	67.03
	Deceased	30	32.97
T	T1	51	56.04
	T2	9	9.89
	T3	27	29.67
	T4	1	1.10
	Unknown	3	3.30
N	N0	79	86.81
	N1	2	2.20
	NX	10	10.99
M	M0	81	89.01
	M1	9	9.89
	MX	1	1.10
Fuhrman grade	G1	13	14.29
	G2	48	52.75
	G3	15	16.48
	G4	14	15.38
	Unknown	1	1.10

### Application of the mPS system to ccRCC subsets

We further investigated whether mPS is also applicable to distinguish samples with different prognosis in different ccRCC subsets in TCGA data set. The results indicated that there was a significant difference regarding the prognosis among different mPS groups of patients in low-, median- and high-mPS groups at the clinical T1 and T2 stage in TCGA cohort (Figure 4C). Meanwhile, we also examined the utility of mPS for different TNM tumor stages determined from clinical information in ICGC cohort. The results suggested that the mPS system also stratified OS of patients in low- and high-mPS groups at the clinical T1 and T2 stage in the ICGC cohort (Figure 4D). We thus conclude that the mPS system can further stratify different ccRCC subsets in different ccRCC cohorts.

### Univariate and multivariate Cox regression of mPS

To determine whether the prognostic ability of mPS for survival prediction was independent from other clinical parameters as a prognostic factor for ccRCC, we carried out univariate Cox regression analysis and multivariate Cox regression analysis on mPS and other clinical factors (age, gender, grade, stage, metastasis, lymph node, hemoglobin, platelet, and calcium result). The univariate analysis revealed that the variables of age, histological grade, stage, metastasis result, lymph node result, platelet result, hemoglobin and mPS score were significantly related to the prognosis of ccRCC patients (*P*<0.05). When entered into the significant factors into multivariate Cox regression analysis, the mPS score (HR=0.46, *P*<0.001), metastasis result (HR=1.83, *P*=0.002), platelet result (HR=1.74, *P*=0.016), hemoglobin result (HR=1.60, *P*=0.007), age (HR=1.58, *P*=0.003) and stage (HR=2.18, *P*=0.025) were found to be independently associated with OS in the entire TCGA cohort (Table 4). These results demonstrated that the mPS system could be applied independently to predict the survival outcomes of ccRCC patients.

**Table 4:** Univariate and multivariate analyses of OS in the TCGA cohort.

**Variables**		**Univariate analysis**			**Multivariate analysis**		
		**HR**	**95%CI**	***P***	**HR**	**95%CI**	***P***
mPS	High vs. Low	0.31	0.22-0.42	<0.001	0.46	0.32-0.65	<0.001
Grade	3&4 vs. 1&2	2.60	1.85-3.67	<0.001	1.41	0.98-2.02	0.07
Gender	Male vs. Female	0.94	0.69-1.28	0.700			
Hemoglobin	Low vs. Normal	2.37	1.66-3.39	<0.001	1.60	1.14-2.26	0.007
Age	Older vs. Younger	1.70	1.26-2.29	<0.001	1.58	1.17-2.14	0.003
Metastasis	M1 vs. M0	4.33	3.17-5.92	<0.001	1.83	1.24-2.70	0.002
Lymph node	N1 vs. N0	3.43	1.82-6.46	0.001	1.30	0.67-2.50	0.44
Stage	III&IV vs. I&II	3.93	2.86-5.39	<0.001	2.18	1.10-4.32	0.025
T stage	T3&T4 vs. T1&T2	3.21	2.39-4.35	<0.001	0.89	0.49-1.62	0.72
Calcium	Low vs. Normal	0.82	0.57-1.16	0.300			
Platelet	Elevated vs. Normal	3.76	2.49-5.68	<0.001	1.74	1.11-2.71	0.016

### Establishment of the nomogram model for survival prediction

**Figure 5:**
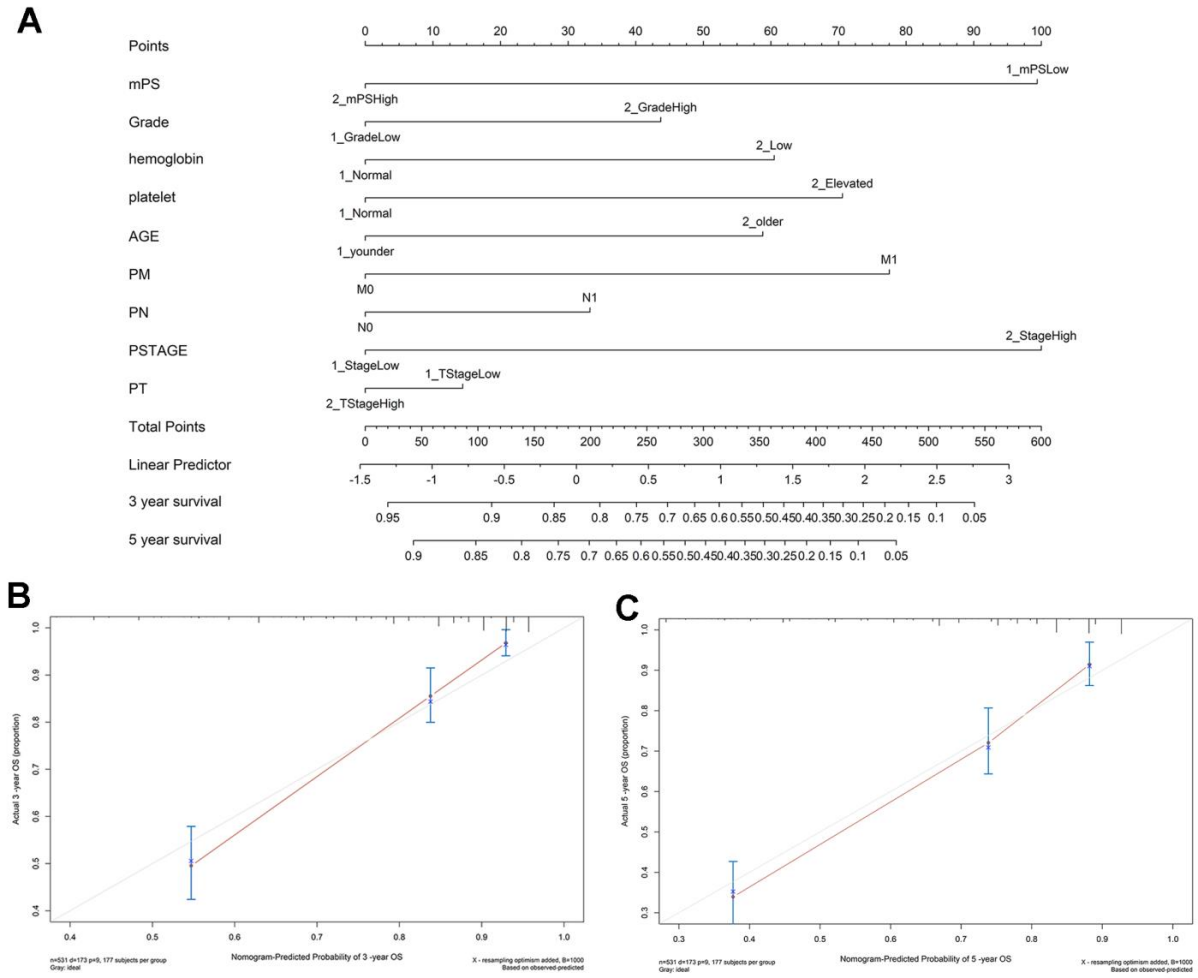
**Nomogram construction results.** (**A**) Nomogram to predict the 3- and 5-year OS for ccRCC patients in the TCGA cohort; (**B**, **C**) calibration curves for the nomogram model of the 3- and 5-year OS.

In order to gain an effective tool to predict the survival probability with ccRCC patients, a nomogram was constructed based on mPS and clinical information using the TCGA cohort. After integrating all significant independent variables, the final model was established and presented as a nomogram to estimate the probability of the 3- and 5-year OS for ccRCC (Figure 5A). C-index was calculated to assess the predictive value of the model. The nomogram had a C-index of 0.79, which revealed relatively high discrimination ability. In addition, the calibration plots exhibited optimal agreement between the model prediction and actual observation for predicting ccRCC survival probability at 3- and 5- year (Figure 5B, 5C).

### Gene set enrichment analysis-based KEGG analysis

**Figure 6:**
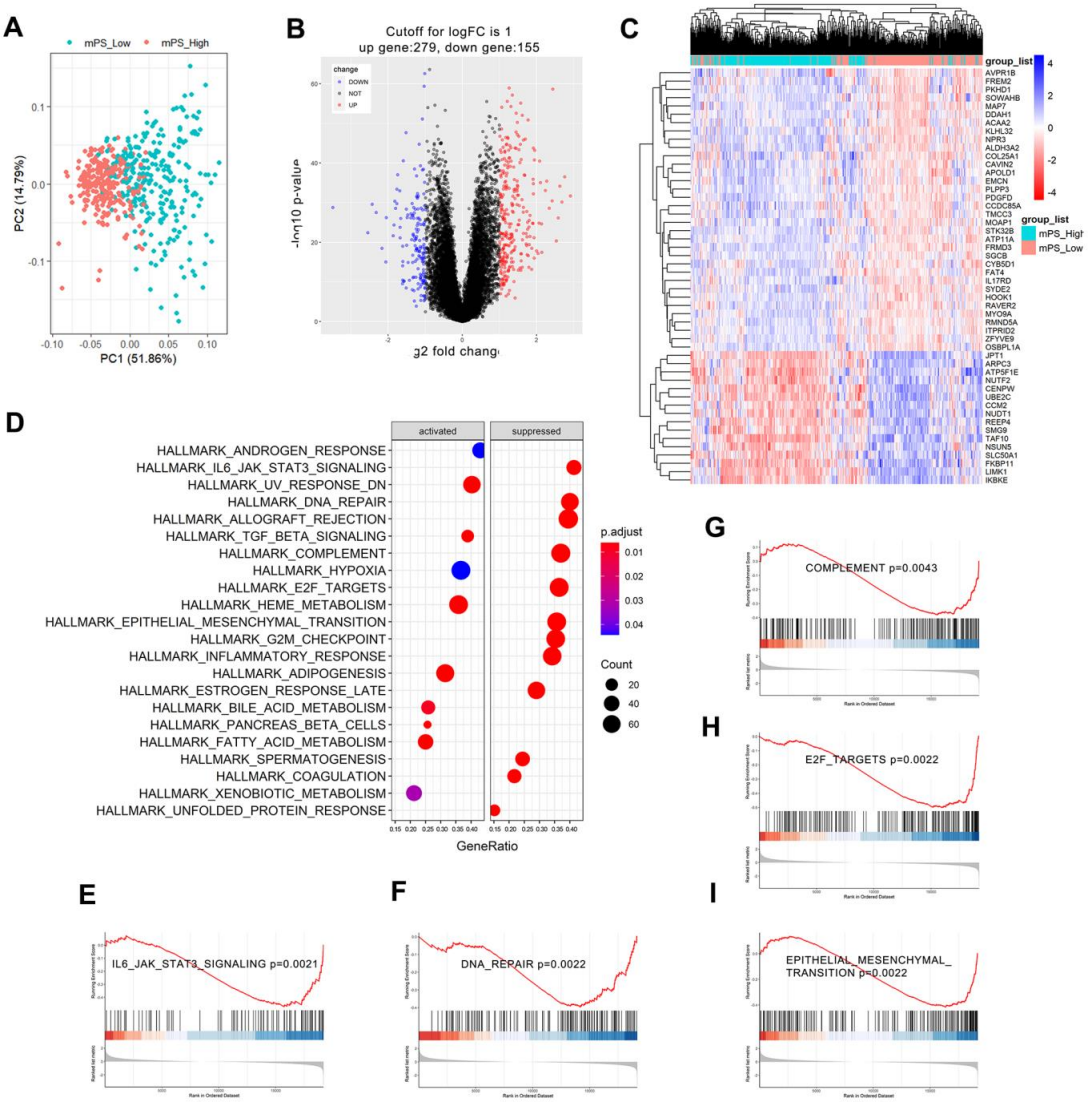
**GSEA analysis results of differentially expressed genes between low- and high-mPS groups.** (**A**) Principal component analysis result; (**B**) Volcano plot of the differentially expressed genes; (**C**) The heat map of the top 50 differentially expressed genes; (**D**) Significantly enriched activated and suppressed KEGG pathways. The vertical items are the names of KEGG terms, and the length of horizontal graph represents the gene ratio. The depth of the color represents the adjusted p-value. The area of circle in the graph means gene counts. (**E**–**I**) GSEA-based KEGG-enrichment plots of representative gene sets from activated and suppressed pathways: “IL6/JAK/STAT3 signaling” (**E**), “DNA repair” (**F**), “complement” (**G**), “E2F targets” (**H**), and “epithelial mesenchymal transition” (**I**).

Based on the Limma R package, a total of 434 genes were identified to differentially express between low- and high-mPS groups, of which 279 genes were up-regulated and 155 genes were down-regulated in high-mPS group compared with low-mPS group. The principal component analysis result was plotted at Figure 6A while the volcano plot of the differentially expressed genes was illustrated at Figure 6B. In addition, the heat map of the top 50 differentially expressed genes was presented at Figure 6C. The differentially expressed genes were then selected for Gene set enrichment analysis (GSEA) analysis. As a result, a total of 20 prominent Kyoto Encyclopedia of Genes and Genomes (KEGG) pathways including activated and suppressed pathways were selected (Figure 6D). Activated pathways mainly includes ultraviolet (UV) response, transforming growth factor-beta (TGF-beta), heme metabolism, adipogenesis, and bile acid metabolism while suppressed pathways were mainly concentrated on IL6/JAK/STAT3 signaling, DNA repair, allograft rejection, complement, E2F targets, epithelial mesenchymal transition, G2M checkpoint, and inflammatory response. GSEA enrichment plots of representative gene sets on “epithelial mesenchymal transition”, “IL6/JAK/STAT3 signaling”, “complement”, “E2F targets”, and “DNA repair” were also shown in Figure 6E–6I.

### Landscape of mutation profiles

**Figure 7:**
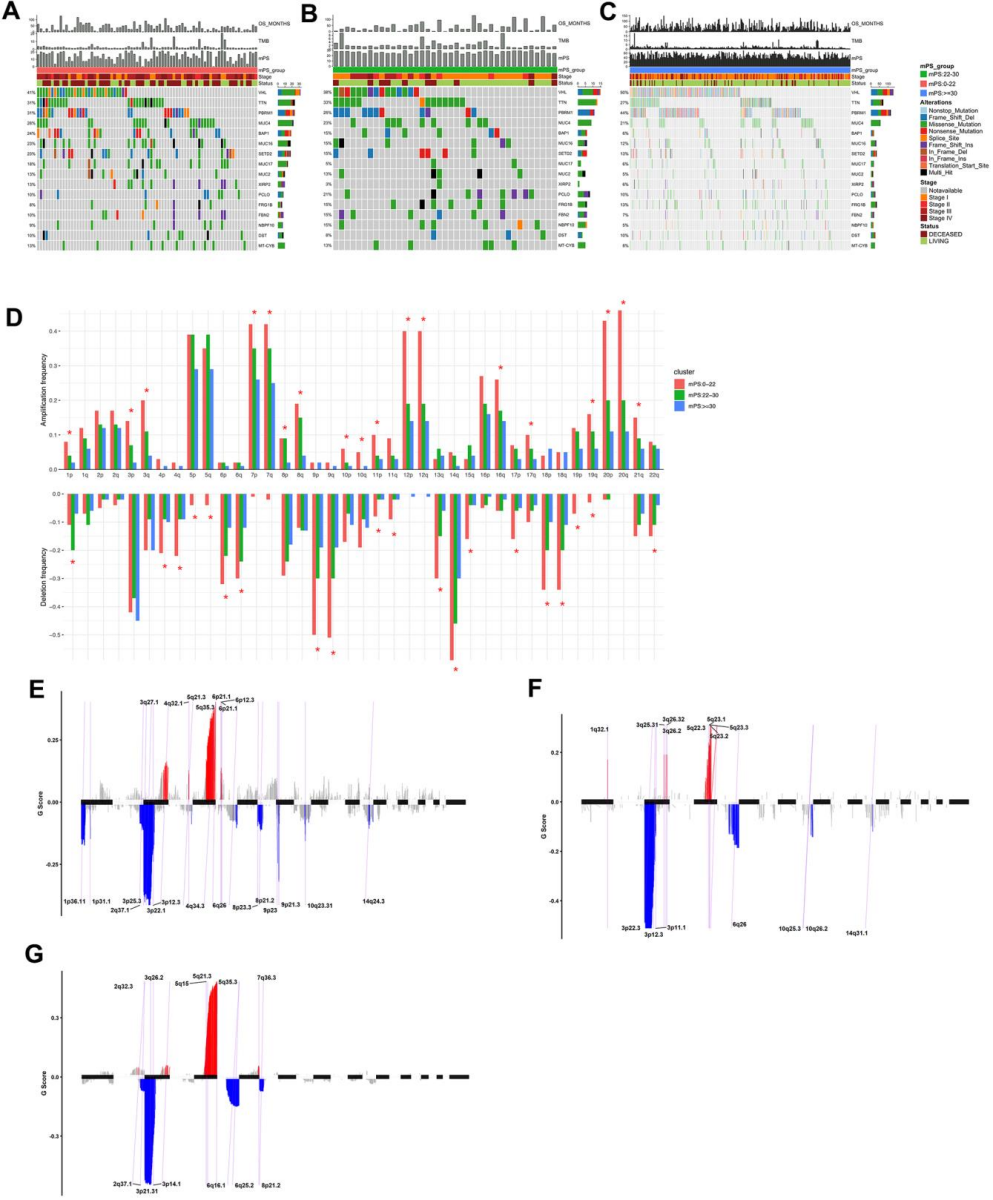
**Mutations and copy number variations in the three subgroups stratified by mPS system.** (**A**–**C**) Distribution and phenotype of common gene mutations in the three subgroups stratified by mPS system: (**A**) low-mPS group (mPS<22); (**B**) median-mPS group (22≤mPS<30); (**C**) high-mPS group (mPS>30). (**D**) The levels of amplification and deletion of chromosome arms of the three subgroups stratified by mPS system. Objects with pentagrams indicate there is a significant difference in the frequency distribution among the three subgroup (P < 0.01); (**E**–**G**) The copy number variations located on minimal common regions (MCRs) of the three subgroups stratified by mPS system: (**E**) low-mPS group (mPS<22); (**F**) median-mPS group (22≤mPS<30); (**G**) high-mPS group (mPS≥30).

The somatic mutation profiles of 531 ccRCC patients were also downloaded from TCGA. According to the above analysis, the TCGA ccRCC samples can be divided into three groups, namely low-mPS group (mPS<22), median-mPS group (22≤mPS<30), and high-mPS group (mPS≥30), and the number of samples in these three groups were 129, 55, 347, respectively. The “maftools” package was applied to visualize the results according to the mutation data with VCF format. The statistically significant mutated genes were identified. Here we mainly focused on the top ten significantly mutated genes for in-depth analyses. A total of 16 significantly mutated genes were then retrieved after taking the combination of the top 10 mutated genes in the three groups. The mutation distributions and annotations of the identified 16 genes in these three groups of samples were illustrated in waterfall plot (Figure 7A–7C). The results indicated that there were significant differences regarding the mutations among the three groups stratified by mPS system, which may help us understand the promising utility of mPS to some degree.

### Copy number variation analysis

The segmented CNVs in the three subgroups of ccRCC samples were applied to screen the significantly frequent CNVs by the Genomic Identification of Significant Targets in Cancer (GISTIC) method. Specifically, in the low-mPS subgroups (mPS<20), the amplifications of 7q, 7p and deletions of 9p, 9q were identified more significant. In addition, in the median-mPS subgroups (22≤mPS<30), the amplifications of 5q, 5p and deletions of 3p, 4q were considered statistically significant. Moreover, in the high-mPS subgroups (mPS≥30), frequent amplifications were observed in chromosomal arms 7q, and 7p, and deletions were observed in 9p, and 9q. Chi-square test was performed on the frequency distribution of each chromosome arm. The frequency distribution of amplification and deletion of chromosome arms in the three groups of samples obtained by GISTIC analysis were presented at Figure 7D.

Then, the CNVs located on minimal common regions (MCRs) were detected in the three subgroups of ccRCC samples (Figure 7E–7G). In the low-mPS subgroups, 26 MCRs of CNVs were identified including 11 amplifications and 15 deletions. The most significant gained regions were located at 5q35.3, while the most significant regions of loss were located at 3p22.1 and 3p25.3. The distribution of these MCRs in the genome was shown in Figure 7. In the median-mPS subgroups, a total of 8 amplifications and 8 deletions of MCRs were detected. In detail, regions 5q23.3 and 5q23.2 with amplifications and 3p22.3, 3p12.3 with deletion were the most frequently altered sites. Moreover, a total of 6 amplifications and 6 deletions of MCRs were detected in the high-mPS subgroups. The most significant amplification regions were 5q35.3, 5q21.3, etc., while the most significant deletion regions were 3p21.31, 3p14.1, etc. Collectively, these findings suggested that the level of chromosomal amplification and deletion was correlated with the mPS score and prognosis, as a worse outcome subgroup (low-mPS subgroup) has more genomic abnormality compared with other groups. In general, a worse outcome may be correlated with more genomic abnormality, revealing that the chromosomal amplification and deletion identified by mPS system can be a patient stratification for immune checkpoint therapy.

### Immune cell infiltration in the tumor microenvironment

**Figure 8:**
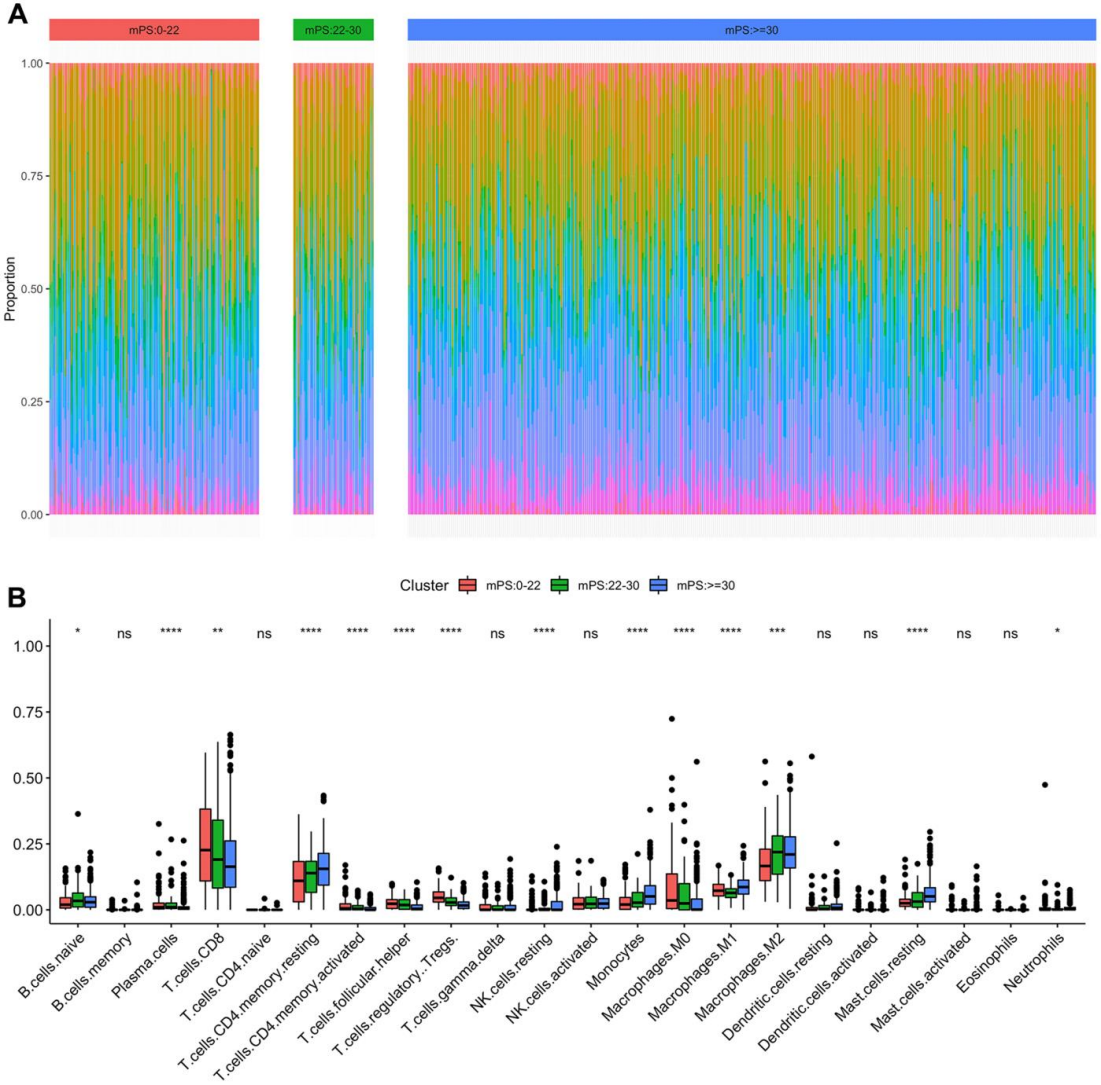
**Immune subtypes in patients with ccRCC.** (**A**) Unsupervised clustering of all samples based on immune cell proportions in low-, median- and high-mPS groups; (**B**) 22 types of adaptive and innate immune cells in low-, median- and high-mPS groups (ns, P>0.05; *,P<0.05; **, P<0.01; ***, P<0.001; ***, P<0.0001;by unpaired two-tailed t test).

Immune cell infiltration has been recognized as one of the most important factors that may positively or negatively shape tumor initiation, progression, or survival outcome. In this study, we analyzed the composition of tumor-infiltrating immune cells in ccRCC samples based on the RNA-seq data of ccRCC samples in TCGA using CIBERSORT. When characterizing the abundances of different immune cell types with CIBERSORT, 22 subsets of tumor-infiltrating immune cells in 531 samples were obtained at a threshold of *P*-value < 0.05, including memory B cells, activated dendritic cells, and M0 macrophage. The distribution results of the inferred fractions of immune cell populations in different samples produced by CIBERSORT were illustrated at Figure 8A. In addition, Figure 8B depicted the differences of the ratio distributions of 22 infiltrating immune cells in the three subgroups of samples including low-mPS, median-mPS, and high-mPS group. The significant immune gene signatures among the three groups included signatures for naive B cells, plasma cells, CD8 T cells, CD4 memory resting T cells, CD4 activated memory T cells, follicular helper T cells, regulatory tregs T cells, resting NK cells, monocytes, M0 macrophages, M1 macrophages, M2 macrophages, resting mast cells, and neutrophils, suggesting that considerable variability existed in the nature of the tumor immune infiltrate across different subgroups of ccRCC stratified by the mPS system, partly accounting for molecular features of the tumor underlying mPS system.

## DISCUSSION

Although the advances of diagnostic techniques and comprehensive treatment have enhanced the survival and life quality of ccRCC patients to some extent, about 30% of patients undergoing curative nephrectomy still developed recurrence and metastasis when the clinical outcomes are poor. In recent years, the utilization of high-throughput sequencing technology has brought up a series of genome-wide biomarkers for ccRCC. However, they are still not enough for accurate prediction and individualized treatment. Therefore, exploring prognostic tools to more accurately manage patients with ccRCC with poor survival is becoming increasingly significant. In this study, we developed a new molecular prognostic signature (mPS system) to predict the overall survival of ccRCC patients.

We first identified 275 differentially expressed genes which were associated with the prognosis of ccRCC. We then used two computational learning models (neural network and random forest) to further screen 21 genes for establishing the mPS system, which combined gene expression level, HR value and gene weight. The survival analysis indicated that there was a significant difference among the three subgroups stratified by the mPS system. Moreover, the prognostic value of the mPS system was validated in independent ccRCC cohorts. Besides, we confirmed that the mPS system is also applicable to distinguish samples with different prognosis in different ccRCC subsets. In our study, we demonstrated that the mPS system may offer valuable information to clinicians regarding variables that are the most useful for patient stratification.

As we all know, ccRCC is a heterogeneous tumor with various confounding factors involved in the initiation and progression of this disease. In this study, univariate and multivariate Cox regression analyses were conducted to assess the correlation between several clinical factors, along with mPS and survival outcome of ccRCC patients. The results showed that mPS score and several clinical factors were independent prognostic factors in patients with ccRCC. Afterwards, a promising clinicopathologic prognostic nomogram was constructed by modeling mPS score and significant factors to predict the 3-, and 5-year OS for patients with ccRCC. Nomograms have frequently served as a vital component of modern medical decision-making under complicated clinical conditions without needing standard guidelines [12]. They can make the results of prediction models clear by presenting visual graphical interfaces, thus providing an opportunity to assess various patient-based variables and predicting a patient’s individualized risk for survival or a specific outcome [13]. Our developed nomogram has indicated moderately high discrimination and sufficient calibration. We suggest using this nomogram to further improve the personalized management of ccRCC patients.

After reviewing the existing literatures, we found that these twenty-one genes are more or less related to tumors. Among them, ten genes were identified directly associated with ccRCC based on PubMed literature reports. For example, Semaphorin 3G (SEMA3G) has been identified as an immune-related gene which is associated with the prognosis of ccRCC patients [14, 15]. Pituitary tumor-transforming gene 1 (PTTG1) is a recently identified oncogene involved in the progression of malignant tumors, and the expression of the PTTG1 oncogene has been confirmed to be related to progression and prognosis of ccRCC patients [16]. There is growing evidence that Claudin 4 (CLDN4) plays a significant role in the occurrence and development of chromophobe renal cell carcinoma and could serve as an independent risk biomarker for the overall survival in patients with chromophobe renal cell carcinoma [17]. Zinc finger protein 395 (ZNF395), a ccRCC master regulator, was identified as a downstream protein of CCDC50-S, and the interaction initiated oncogenic pathways which were highly associated with the pathogenesis of ccRCC [18]. Apelin receptor (APLNR) expression was negatively related to PD-L1 expression by tumor cells in a subset of patients with ccRCC and the expression of APLNR has been recognized as an independent prognostic factor for survival of patients with ccRCC [19]. Recent evidence has identified Ubiquitin conjugating enzyme E2 C (UBE2C) as a pathological stage-relevant gene, which is associated with carcinogenesis and progression in ccRCC, and may provide potential diagnostic, therapeutic and prognostic biomarkers for ccRCC [20]. Previous studies have indicated Cadherin 4 (CDH4) was significantly linked with the survival outcome of ccRCC patients and the transcriptional level of CDH4 may serve as an effective diagnostic and prognostic biomarker for ccRCC patients [21]. Recent findings reveal that G protein subunit gamma 7 (GNG7) is a tumor suppressor in ccRCC progression and has a potential to serve as a new biomarker or therapeutic target in ccRCC [22]. CEACAM1 has been known as a tumor suppressor in various epithelial tumors. Transient expression of CEACAM1 by tumor cells and subsequent homophilic interaction with CEA cell adhesion molecule 1 (CEACAM1) on tumor-infiltrating lymphocytes may provide a novel immune escape mechanism in ccRCC. Moreover, Plasminogen activator, urokinase receptor (PLAUR) was developed and validated as a prognostic immune-associated gene signature in ccRCC along with other six genes [23]. However, eleven genes (CRABP2, DDAH1, TGFA, SPATA18, SCGN, CYP39A1, IL15RA, APOLD1, NTN4, PABPC1L, and SIM2) have not been studied in relation to ccRCC, according to PubMed searches for “GENE and ccRCC”. Further basic and clinical studies are required to validate our observations and the mechanisms underlying the prognostic value of 21 genes on which mPS is based and for the development of novel therapeutic targets to prolong OS of ccRCC patients.

Recently, AI-based algorithms have been successfully applied in medical fields as they are capable of learning patterns from massive, complex datasets and creating useful predictive outputs [24]. Moreover, AI-based technologies are well adapted to rapid, high-volume data processing, promoting tailored and specific management according to each patient’s characteristics [25]. In particular, artificial neural networks are computational models inspired by biological neural networks, which are currently most common practiced models of AI-based algorithms developed for survival prediction and decision-making [26]. Another AI technique is the random forest tool, which is a multivariate prediction model that conducts a computationally extensive and robust data-mining and could handle large sets of proposed variables as inputs for screening variables related to the outcomes of interest [27]. Recently, Nakayama et al. established a universal mPS system that relies on the expression status of 23 genes by using AI models (random forest and neural network), which is applicable to almost all subsets of breast cancer patients [28]. Moreover, Aramini et al. set up a mPS system for stratifying early-stage, resected lung cancer patients, which could be used for predicting risk of distant recurrence and informing treatment and surveillance decisions [29]. In our study, we developed the mPS system based on two computational learning models (AI-based random forest and neural network), which has exhibited promising predictive performance for ccRCC patients.

Based on the mPS system, we preliminarily divided ccRCC patients into low- and high-mPS groups. Then, differentially expressed genes between these two groups were identified and used for GSEA analysis. A series of activated and suppressed pathways were identified including “epithelial mesenchymal transition”, “IL6/JAK/STAT3 signaling”, “complement”, “E2F targets”, and “DNA repair”, which are highly involved in the initiation and progression of ccRCC. For example, the epithelial mesenchymal transition (EMT) has now been recognized as a complicated biological trans-differentiation process, promoting epithelial cells to transiently gain mesenchymal features, containing motility and metastatic potential [30]. EMT has been highly involved in a extensive range of malignant tumor types including ccRCC and can be driven by a conserved set of inducing signals, transcriptional regulators and downstream effectors [31]. IL-6/JAK/STAT pathway is aberrantly hyper-activated in many types of cancer including ccRCC and plays a significant part in the progression of cancer cachexia through regulating the inflammatory response [32]. Studies have convinced the roles of DNA repair deregulation in promoting immune recognition and immune destruction of cancer cells [33]. Emerging evidence has identified that defects in DNA repair machinery play an important part in the pathogenesis and progression of human cancer [34]. Moreover, the information gathered so far indicated that E2F is one of the most important transcription factors that regulates various cellular functions related to cell cycle and apoptosis and involves not only in proliferation and tumorigenesis but also in apoptosis and differentiation [35]. These pathways may provide new insights for the occurrence and development of ccRCC.

In recent years, it is widely acknowledged that genetic or epigenetic alterations play an important role in a variety of transcriptional and non-transcriptional biological processes [36]. Mutations and CNVs have been vital forms of epigenetic modifications, which can cause genomic and molecular phenotype heterogeneity, promoting the initiation and progression of complex diseases, including cancer [37, 38]. In this study, we presented an integrative analysis of multiple types of genomic data, including mutations, and CNVs. We identified 16 significantly mutated genes, the mutation distributions and annotations of which were statistically different in the three groups stratified by mPS system. Moreover, the significantly frequent CNVs in the mPS subgroups of ccRCC samples were identified by GISTIC. In addition, the CNVs located on MCRs were also detected in the three subgroups of ccRCC samples. The results revealed that the CNVs were significantly different among different mPS subgroups. Moreover, a worse outcome is correlated with more genomic abnormality. These findings further supported the promising predictive value of mPS system and provided potential therapeutic targets for ccRCC patients.

Increasing evidence supports the importance of the immune infiltration of tumors on the initiation and progression of various tumors, which could provide potential biomarkers to improve the reliability and precision of diagnosis and prognosis [39]. Recent studies have extensively investigated the dysfunction of immune cell infiltration in promoting the metastasis and invasion of ccRCC through mediating tumor cell budding and disruption of focal basal cell layer [40]. In this study, we applied the newly developed algorithm CIBERSORT to estimate different expressional cell patterns of immune infiltration in different mPS subgroups. The distributions of immune cell fractions in ccRCC tissues were significantly different among different subgroups stratified by the mPS system. Several important immune cells were identified differentially expressed among the mPS subgroups, including naive B cells, CD8 T cells, CD4 memory resting T cells, CD4 activated memory T cells, which may explain the prognosis differences of the low-mPS, median-mPS, and high-mPS group.

The advantage of this study is that, firstly, we developed a simple and cost-effective mPS system by using multivariate models including neural network and random forest, which were beneficial to obtain a stable scoring system. Secondly, the scoring model was verified in the ICGC data set, and this scoring model is also effective for subgroups. Moreover, the sub-population samples were further characterized, adding mutation analysis, CNV analysis, and immune infiltrating cell ratio analysis. The mutations, CNVs and tumor immune infiltrations will provide new insights into carcinogenesis and might promote to identify potential therapeutic targets for ccRCC. We also combined the mPS system with several independent variables into a predictive nomogram, which could provide convincing information for helping clinicians to illustrate the prognosis of patients with ccRCC.

Nevertheless, there are inevitably several limitations of our study that should be acknowledged. To begin with, the number of samples with follow-up data of the validation cohort was limited due to availability, which limits the ability to adjust for confounding factors. Thus, additional large validation datasets are required to further improve prediction performance of the mPS system. Then, we were unable to compare the prognostic value of mPS with that of other representative models. Furthermore, these results from our study were provided through analytic methods and machine learning without experimental validation. Thus, it is of great importance to find the molecular mechanism for further characterization of mPS.

## CONCLUSIONS

In summary, we developed a new molecular prognostic model with AI-based methods, called the mPS system, based on expression levels of twenty-one prognostic genes. The mPS system obtained from AI-based method can be used as a medical decision support system that may provide critical information for prognostic assessment of patients with ccRCC before initial intervention. In addition, we explored the potential mechanisms underlying the mPS system by performing GSEA analysis and evaluating the differences of mutations, CNVs and immune cell infiltrations among the subgroups stratified by the mPS system, which should be validated to further characterize the molecular background of the mPS system.

## MATERIALS AND METHODS

### Data source

The mRNA expression data of TCGA ccRCC discovery cohort (comprising 72 normal specimens and 539 ccRCC specimens) were retrieved from the UCSC Xena database (https://xenabrowser.net/datapages/) [41]. Clinical data pertaining to patients’ age, gender, grade, stage, survival outcome were also acquired from the TCGA data portal by using cgdsr package in R, which contained clinical information of 531 ccRCC patient samples. We downloaded 523 SNP6 copy number samples with corresponding transcriptome data from http://firebrowse.org/. Moreover, the R-based TCGAbiolinks was employed to collect the SNP data of ccRCC, which consists of 380 samples with corresponding transcriptome data. We adopted another ccRCC data set from the ICGC database as the validation cohort, which covers 91 ccRCC samples with mRNA expression and clinical data [42].

### Identification of prognosis-associated genes and differential expression analysis

The public data were retrieved from https://xenabrowser.net/datapages/. All human protein-coding genes were assessed the potential utility as a prognostic predictor with the TCGA ccRCC discovery cohort. The K-M method was performed for survival analysis by using R package survival and survminer [43]. According to the median value of gene expression, the ccRCC samples were divided into high or low expression group for a particular gene. The cox regression analysis in the survival package was applied to calculate the HR and 95% confidence interval (CI) of each prognosis-related gene. LIMMA R software package was used to analyze differential expressed genes [44]. The adj.*P*-value <0.05 and a |log2FC| >2 were regarded as the cutoff values of statistical significance.

### Random forest prediction model

Random forest is one of the most widely used nonparametric techniques for data classification and regression analysis [45]. The idea of random forest is to establish multiple trees that use randomly generated samples from existing situations with a bootstrapping technique and to generate an average prediction of the individual trees [46]. The basic unit of random forest is a decision tree. To classify a sample, each tree in the forest is given a random input vector and all vectors in the random forest are independent and identically distributed. Random forest is designed to randomize the column variables and row observations of the dataset, generate multiple classification subgroups, and finally summarize the classification results. In this study, we applied the R package randomForest to build the random forest prediction model by using the ccRCC samples in TCGA. The expression status of 275 selected prognostic-related differentially expressed genes was used as the model input, while the survival status of the sample in the third year (alive=0, deceased=1) was selected as the output, where ntree was set to 500 and maximum depth (max-depth) was set to 10. To assess the generalized performance, we applied a stratified 10-fold cross-validation test (CV = 10). We measured the importance of each feature by referring to the average decrease value of node impurity: the larger the value, the greater importance of the variable. After a 10-fold cross-validation, we identified 21 genes on the basis of feature importance values for further artificial neural network mode (cutoff=0.5).

### Artificial neural network mode

We first transformed the expression status of the 21 genes to “Gene-Score” based on the expression level (above or below the median) and integrated HR for each gene with the following step function:

**Table:** 

**Gene-score matrix**		**Gene expression**	
		**low**	**high**
Integrated HR	<1	1	0
	>1	0	1

Subsequently, an artificial neural network system was constructed and trained. At each hidden neuron node, the ReLU (rectified linear unit) was applied as the activation function [47]. In the output layer, two nodes were set including a_1_ and a_2_, which represented alive and deceased, respectively. We employed a softmax function for each node, and designated y_2_ (probability of death; that is, the a_2_ node) as Y. We applied cross entropy error for the model loss function (E). Moreover, we used the Adam method for the optimization of each weight (learning rate=0.001; epochs=1000). After the neural network model was constructed, the weight of each node in the input layer (Gene-Weight) was used to calculate mPS (the sum of Gene-Score*Gene-Weight for all 21 genes). In our study, the Python-based Keras library was applied for neural network training.

### Univariate and multivariate Cox regression analysis

Univariable and multivariable Cox regression analyses were applied to evaluate the associations between overall survival and potential confounders including mPS and other clinical variables [48]. Univariable Cox regression analysis was performed to explore the potential confounders. Afterwards, significant prognostic variables with a *P*-value < 0.05 in univariate analysis were further entered into a multivariate Cox proportional hazards model along with the corresponding 95% CI for each potential risk factor. *P*-values less than 0.05 were considered statistically significant.

### Construction of prognostic nomogram

A nomogram was established to predict 3- and 5-year OS by including all independent prognostic factors of the multivariate Cox regression analysis by using RMS package in the R Statistical [49]. Afterwards, we illustrated the predicted and observed results in the calibration curves for the visualization of the predictive performance of the nomogram. The discrimination of the prognostic models was estimated and compared by Harrell’s concordance index (C-index). A higher C-index indicated a superior discriminative capacity for prognostic prediction. Generally, a C-index value ≥0.70 suggests a good fit [50].

### Gene set enrichment analysis

GSEA was applied to further understand mPS-associated pathways [51]. The ccRCC patients in the TCGA cohort were divided into low- and high-mPS groups based on the median expression value of mPS. Then, the genes differentially expressed between low- and high-mPS groups were identified based on Student’s t-test in the Limma R package. The *P*-value < 0.05 was regarded as the cutoff value of statistical significance. Afterwards, GSEA was performed based on GSEABase package on the R platform.

### Mutational pattern analysis

The mutation data of ccRCC sample in TCGA were processed and visualized by R package maftools. Maftool is an efficient and comprehensive tool, which provides various analysis and visualization modules that are frequently applied in cancer genomic studies, such as driver gene identification, pathway, signature, enrichment, and association analyses [52]. The top 10 frequently mutated genes of each group were identified and compared by performing Chi-square test. Tumor mutation burden (TMB) was defined as the total amount of coding errors of somatic genes, base substitutions, insertions or deletions detected across per million bases [53]. In the present study, the mutation frequency was calculated with number of variants/the length of exons (45 million) for each sample.

### Copy number variations analysis

Loci of amplification and deletion were investigated across samples to define MCRs targeted by overlapping events in two or more. In our study, we used the GISTIC method to detect the common CNV regions in all samples based on SNP6 Copy Number segment data, including CNVs at the chromosomal arm level and MCRs between samples [54]. GISTIC is an algorithm designed to distinguish regions of variation that are more possible to trigger cancer pathogenesis. We chose the threshold of *q*-value<0.1 for the MutSigCV method. In addition, we selected the confidence level of 0.95 when the peak interval was determined. Moreover, the area (greater than 0.98) was applied as the standard when analyzing the variation of the chromosomal arm level. We performed these analyses by using the MutSigCV module in the online GenePattern analysis tool (https://cloud.genepattern.org/gp/pages/index.jsf), which is developed by Broad Research Institute [55].

### Evaluation of immune cell infiltration

To quantify the proportions of immune cells among different subtypes of ccRCC, the CIBERSORT algorithm was applied, which is a deconvolution method that uses a series of reference gene expression values for a minimal representation of each cell type and investigates the cell component of complex tissues according to the gene expression data from massive tumor samples with support vector regression [56]. The normalized 531 ccRCC TCGA RNA-seq data were uploaded to the CIBERSORT web portal (http://cibersort.stanford.edu/), running with the 1000 permutations and LM22 signature, which defines 22 immune cell subtypes including B cells, T cells, natural killer cells, dendritic cells, macrophages, and myeloid subsets. At a threshold of *P* < 0.05, the results of the inferred fractions of immune cell populations identified by CIBERSORT were represented to be statistically significant. For each sample, the final CIBERSORT output estimates were normalized and immune cell type fractions summed up to one.

### Ethical statement

All information in this study was retrieved from public datasets; therefore, written informed consent was not necessary. This study meets the publication guidelines provided by the individual public datasets.

## References

[1] Siegel RL, Miller KD, Jemal A. Cancer statistics, 2020. CA Cancer J Clin. 2020; 70:7–30. 10.3322/caac.21590 31912902

[2] Hsieh JJ, Purdue MP, Signoretti S, Swanton C, Albiges L, Schmidinger M, Heng DY, Larkin J, Ficarra V. Renal cell carcinoma. Nat Rev Dis Primers. 2017; 3:17009. 10.1038/nrdp.2017.9 28276433 PMC5936048

[3] Barata PC, Rini BI. Treatment of renal cell carcinoma: current status and future directions. CA Cancer J Clin. 2017; 67:507–24. 10.3322/caac.21411 28961310

[4] Adashek JJ, Salgia MM, Posadas EM, Figlin RA, Gong J. Role of biomarkers in prediction of response to therapeutics in metastatic renal-cell carcinoma. Clin Genitourin Cancer. 2019; 17:e454–60. 10.1016/j.clgc.2019.01.004 30733185

[5] Linehan WM, Ricketts CJ. The cancer genome atlas of renal cell carcinoma: findings and clinical implications. Nat Rev Urol. 2019; 16:539–52. 10.1038/s41585-019-0211-5 31278395

[6] Ran L, Liang J, Deng X, Wu J. miRNAs in prediction of prognosis in clear cell renal cell carcinoma. Biomed Res Int. 2017; 2017:4832931. 10.1155/2017/4832931 29392135 PMC5748131

[7] Dizman N, Philip EJ, Pal SK. Genomic profiling in renal cell carcinoma. Nat Rev Nephrol. 2020; 16:435–51. 10.1038/s41581-020-0301-x 32561872

[8] Schwalbe N, Wahl B. Artificial intelligence and the future of global health. Lancet. 2020; 395:1579–86. 10.1016/S0140-6736(20)30226-9 32416782 PMC7255280

[9] Bates DW, Auerbach A, Schulam P, Wright A, Saria S. Reporting and Implementing Interventions Involving Machine Learning and Artificial Intelligence. Ann Intern Med. 2020; 172:S137–44. 10.7326/M19-0872 32479180

[10] Goecks J, Jalili V, Heiser LM, Gray JW. How machine learning will transform biomedicine. Cell. 2020; 181:92–101. 10.1016/j.cell.2020.03.022 32243801 PMC7141410

[11] Rajkomar A, Dean J, Kohane I. Machine learning in medicine. N Engl J Med. 2019; 380:1347–58. 10.1056/NEJMra1814259 30943338

[12] Serenari M, Han KH, Ravaioli F, Kim SU, Cucchetti A, Han DH, Odaldi F, Ravaioli M, Festi D, Pinna AD, Cescon M. A nomogram based on liver stiffness predicts postoperative complications in patients with hepatocellular carcinoma. J Hepatol. 2020; 73:855–62. 10.1016/j.jhep.2020.04.032 32360997

[13] Peng Q, Zhou Y, Jin L, Cao C, Gao C, Zhou J, Yang D, Zhu J. Development and validation of an integrative methylation signature and nomogram for predicting survival in clear cell renal cell carcinoma. Transl Androl Urol. 2020; 9:1082–98. 10.21037/tau-19-853 32676392 PMC7354314

[14] Gao X, Yang J, Chen Y. Identification of a four immune-related genes signature based on an immunogenomic landscape analysis of clear cell renal cell carcinoma. J Cell Physiol. 2020; 235:9834–50. 10.1002/jcp.29796 32452055

[15] Wan B, Liu B, Huang Y, Yu G, Lv C. Prognostic value of immune-related genes in clear cell renal cell carcinoma. Aging (Albany NY). 2019; 11:11474–89. 10.18632/aging.102548 31821170 PMC6932908

[16] Luo Y, Shen D, Chen L, Wang G, Liu X, Qian K, Xiao Y, Wang X, Ju L. Identification of 9 key genes and small molecule drugs in clear cell renal cell carcinoma. Aging (Albany NY). 2019; 11:6029–52. 10.18632/aging.102161 31422942 PMC6738436

[17] Wu H, Fan L, Liu H, Guan B, Hu B, Liu F, Hocher B, Yin L. Identification of key genes and prognostic analysis between chromophobe renal cell carcinoma and renal oncocytoma by bioinformatic analysis. Biomed Res Int. 2020; 2020:4030915. 10.1155/2020/4030915 31998788 PMC6977339

[18] Sun G, Zhou H, Chen K, Zeng J, Zhang Y, Yan L, Yao W, Hu J, Wang T, Xing J, Xiao K, Wu L, Ye Z, Xu H. HnRNP A1 - mediated alternative splicing of CCDC50 contributes to cancer progression of clear cell renal cell carcinoma via ZNF395. J Exp Clin Cancer Res. 2020; 39:116. 10.1186/s13046-020-01606-x 32560659 PMC7304168

[19] Tolkach Y, Ellinger J, Kremer A, Esser L, Müller SC, Stephan C, Jung K, Toma M, Kristiansen G, Hauser S. Apelin and apelin receptor expression in renal cell carcinoma. Br J Cancer. 2019; 120:633–39. 10.1038/s41416-019-0396-7 30783205 PMC6461937

[20] Xu D, Xu Y, Lv Y, Wu F, Liu Y, Zhu M, Chen D, Bai B. Identification of four pathological stage-relevant genes in association with progression and prognosis in clear cell renal cell carcinoma by integrated bioinformatics analysis. Biomed Res Int. 2020; 2020:2137319. 10.1155/2020/2137319 32309427 PMC7142335

[21] Zhou X, Huang H, Cui W, Wang Y, Luo W, Matskova L, Zhou X. Expression and prognostic significance of cadherin 4 (CDH4) in renal cell carcinoma. Med Sci Monit. 2020; 26:e922836. 10.12659/MSM.922836 32511216 PMC7297024

[22] Xu S, Zhang H, Liu T, Chen Y, He D, Li L. G protein γ subunit 7 loss contributes to progression of clear cell renal cell carcinoma. J Cell Physiol. 2019; 234:20002–12. 10.1002/jcp.28597 30945310 PMC6767067

[23] Shen C, Liu J, Wang J, Zhong X, Dong D, Yang X, Wang Y. Development and validation of a prognostic immune-associated gene signature in clear cell renal cell carcinoma. Int Immunopharmacol. 2020; 81:106274. 10.1016/j.intimp.2020.106274 32044664

[24] He J, Baxter SL, Xu J, Xu J, Zhou X, Zhang K. The practical implementation of artificial intelligence technologies in medicine. Nat Med. 2019; 25:30–36. 10.1038/s41591-018-0307-0 30617336 PMC6995276

[25] Topol EJ. High-performance medicine: the convergence of human and artificial intelligence. Nat Med. 2019; 25:44–56. 10.1038/s41591-018-0300-7 30617339

[26] Renganathan V. Overview of artificial neural network models in the biomedical domain. Bratisl Lek Listy. 2019; 120:536–40. 10.4149/BLL_2019_087 31602991

[27] Stephan J, Stegle O, Beyer A. A random forest approach to capture genetic effects in the presence of population structure. Nat Commun. 2015; 6:7432. 10.1038/ncomms8432 26109276

[28] Shimizu H, Nakayama KI. A 23 gene-based molecular prognostic score precisely predicts overall survival of breast cancer patients. EBioMedicine. 2019; 46:150–59. 10.1016/j.ebiom.2019.07.046 31358476 PMC6711850

[29] Aramini B, Casali C, Stefani A, Bettelli S, Wagner S, Sangale Z, Hughes E, Lanchbury JS, Maiorana A, Morandi U. Prediction of distant recurrence in resected stage I and II lung adenocarcinoma. Lung Cancer. 2016; 101:82–87. 10.1016/j.lungcan.2016.09.005 27794412

[30] Yang J, Antin P, Berx G, Blanpain C, Brabletz T, Bronner M, Campbell K, Cano A, Casanova J, Christofori G, Dedhar S, Derynck R, Ford HL, et al, and EMT International Association (TEMTIA). Guidelines and definitions for research on epithelial-mesenchymal transition. Nat Rev Mol Cell Biol. 2020; 21:341–52. 10.1038/s41580-020-0237-9 32300252 PMC7250738

[31] Dongre A, Weinberg RA. New insights into the mechanisms of epithelial-mesenchymal transition and implications for cancer. Nat Rev Mol Cell Biol. 2019; 20:69–84. 10.1038/s41580-018-0080-4 30459476

[32] Johnson DE, O’Keefe RA, Grandis JR. Targeting the IL-6/JAK/STAT3 signalling axis in cancer. Nat Rev Clin Oncol. 2018; 15:234–48. 10.1038/nrclinonc.2018.8 29405201 PMC5858971

[33] Karakaidos P, Karagiannis D, Rampias T. Resolving DNA damage: epigenetic regulation of DNA repair. Molecules. 2020; 25:2496. 10.3390/molecules25112496 32471288 PMC7321228

[34] Cleary JM, Aguirre AJ, Shapiro GI, D’Andrea AD. Biomarker-guided development of DNA repair inhibitors. Mol Cell. 2020; 78:1070–85. 10.1016/j.molcel.2020.04.035 32459988 PMC7316088

[35] Kent LN, Leone G. The broken cycle: E2F dysfunction in cancer. Nat Rev Cancer. 2019; 19:326–38. 10.1038/s41568-019-0143-7 31053804

[36] Mohammad HP, Barbash O, Creasy CL. Targeting epigenetic modifications in cancer therapy: erasing the roadmap to cancer. Nat Med. 2019; 25:403–18. 10.1038/s41591-019-0376-8 30842676

[37] D’Avella C, Abbosh P, Pal SK, Geynisman DM. Mutations in renal cell carcinoma. Urol Oncol. 2020; 38:763–73. 10.1016/j.urolonc.2018.10.027 30478013

[38] Lye ZN, Purugganan MD. Copy number variation in domestication. Trends Plant Sci. 2019; 24:352–65. 10.1016/j.tplants.2019.01.003 30745056

[39] Zhang Y, Zhang Z. The history and advances in cancer immunotherapy: understanding the characteristics of tumor-infiltrating immune cells and their therapeutic implications. Cell Mol Immunol. 2020; 17:807–21. 10.1038/s41423-020-0488-6 32612154 PMC7395159

[40] Zhang S, Zhang E, Long J, Hu Z, Peng J, Liu L, Tang F, Li L, Ouyang Y, Zeng Z. Immune infiltration in renal cell carcinoma. Cancer Sci. 2019; 110:1564–72. 10.1111/cas.13996 30861269 PMC6501001

[41] Goldman MJ, Craft B, Hastie M, Repečka K, McDade F, Kamath A, Banerjee A, Luo Y, Rogers D, Brooks AN, Zhu J, Haussler D. Visualizing and interpreting cancer genomics data via the xena platform. Nat Biotechnol. 2020; 38:675–78. 10.1038/s41587-020-0546-8 32444850 PMC7386072

[42] Zhang J, Bajari R, Andric D, Gerthoffert F, Lepsa A, Nahal-Bose H, Stein LD, Ferretti V. The international cancer genome consortium data portal. Nat Biotechnol. 2019; 37:367–69. 10.1038/s41587-019-0055-9 30877282

[43] Cole SR, Edwards JK, Naimi AI, Muñoz A. Hidden imputations and the kaplan-meier estimator. Am J Epidemiol. 2020; 189:1408–11. 10.1093/aje/kwaa086 32412079 PMC7731992

[44] Ritchie ME, Phipson B, Wu D, Hu Y, Law CW, Shi W, Smyth GK. Limma powers differential expression analyses for RNA-sequencing and microarray studies. Nucleic Acids Res. 2015; 43:e47. 10.1093/nar/gkv007 25605792 PMC4402510

[45] Basith S, Manavalan B, Hwan Shin T, Lee G. Machine intelligence in peptide therapeutics: a next-generation tool for rapid disease screening. Med Res Rev. 2020; 40:1276–314. 10.1002/med.21658 31922268

[46] Luz CF, Vollmer M, Decruyenaere J, Nijsten MW, Glasner C, Sinha B. Machine learning in infection management using routine electronic health records: tools, techniques, and reporting of future technologies. Clin Microbiol Infect. 2020; 26:1291–99. 10.1016/j.cmi.2020.02.003 32061798

[47] Wang D, Zeng J, Lin SB. Random sketching for neural networks with ReLU. IEEE Trans Neural Netw Learn Syst. 2020. [Epub ahead of print]. 10.1109/TNNLS.2020.2979228 32275612

[48] Benítez-Parejo N, Rodríguez del Águila MM, Pérez-Vicente S. Survival analysis and cox regression. Allergol Immunopathol (Madr). 2011; 39:362–73. 10.1016/j.aller.2011.07.007 22014655

[49] Iasonos A, Schrag D, Raj GV, Panageas KS. How to build and interpret a nomogram for cancer prognosis. J Clin Oncol. 2008; 26:1364–70. 10.1200/JCO.2007.12.9791 18323559

[50] Wolbers M, Koller MT, Witteman JC, Steyerberg EW. Prognostic models with competing risks: methods and application to coronary risk prediction. Epidemiology. 2009; 20:555–61. 10.1097/EDE.0b013e3181a39056 19367167

[51] Clark NR, Ma’ayan A. Introduction to statistical methods for analyzing large data sets: gene-set enrichment analysis. Sci Signal. 2011; 4:tr4. 10.1126/scisignal.2001966 21917718 PMC3205944

[52] Mayakonda A, Lin DC, Assenov Y, Plass C, Koeffler HP. Maftools: efficient and comprehensive analysis of somatic variants in cancer. Genome Res. 2018; 28:1747–56. 10.1101/gr.239244.118 30341162 PMC6211645

[53] Galuppini F, Dal Pozzo CA, Deckert J, Loupakis F, Fassan M, Baffa R. Tumor mutation burden: from comprehensive mutational screening to the clinic. Cancer Cell Int. 2019; 19:209. 10.1186/s12935-019-0929-4 31406485 PMC6686509

[54] Sanchez-Garcia F, Akavia UD, Mozes E, Pe’er D. JISTIC: identification of significant targets in cancer. BMC Bioinformatics. 2010; 11:189. 10.1186/1471-2105-11-189 20398270 PMC2873534

[55] Lawrence MS, Stojanov P, Polak P, Kryukov GV, Cibulskis K, Sivachenko A, Carter SL, Stewart C, Mermel CH, Roberts SA, Kiezun A, Hammerman PS, McKenna A, et al. Mutational heterogeneity in cancer and the search for new cancer-associated genes. Nature. 2013; 499:214–18. 10.1038/nature12213 23770567 PMC3919509

[56] Newman AM, Liu CL, Green MR, Gentles AJ, Feng W, Xu Y, Hoang CD, Diehn M, Alizadeh AA. Robust enumeration of cell subsets from tissue expression profiles. Nat Methods. 2015; 12:453–57. 10.1038/nmeth.3337 25822800 PMC4739640

